# How do eHealth Programs for Adolescents With Depression Work? A Realist Review of Persuasive System Design Components in Internet-Based Psychological Therapies

**DOI:** 10.2196/jmir.7573

**Published:** 2017-08-09

**Authors:** Lori Wozney, Anna Huguet, Kathryn Bennett, Ashley D Radomski, Lisa Hartling, Michele Dyson, Patrick J McGrath, Amanda S Newton

**Affiliations:** ^1^ Centre for Research in Family Health IWK Health Centre Halifax, NS Canada; ^2^ Department of Clinical Epidemiology and Biostatistics McMaster University Hamilton, ON Canada; ^3^ Department of Pediatrics Faculty of Medicine & Dentistry University of Alberta Edmonton, AB Canada

**Keywords:** persuasive systems, mental health, Internet-based intervention, review, psychological therapy

## Abstract

**Background:**

Major depressive disorders are common among adolescents and can impact all aspects of their daily life. Traditional therapies, cognitive behavioral therapy (CBT), and interpersonal psychotherapy (IPT) have been delivered face-to-face. However, Internet-based (online) delivery of these therapies is emerging as an option for adolescents. Internet-based CBT and IPT involve therapeutic content, interaction between the user and the system, and different technological features embedded into the online program (eg, multimedia). Studies of Internet-based CBT and IPT for adolescent depression differ on all three aspects, and variable, positive therapy effects have been reported. A better understanding of the treatment conditions that influence therapy outcomes is important to designing and evaluating these novel therapies.

**Objective:**

Our aim was to examine the technological and program delivery features of Internet-based CBT and IPT for adolescent depression and to document their potential relation to treatment outcomes and program use.

**Methods:**

We performed a realist synthesis. We started with an extensive search of published and gray literature. We included intervention studies that evaluated Internet-based CBT or IPT for adolescent depression. We included mixed-methods and qualitative studies, theoretical papers, and policy/implementation documents if they included a focus on how Internet-based psychological therapy is proposed to work for adolescents with depression/depressive symptoms. We used the Mixed-Methods Appraisal Tool to assess the methodological quality of studies. We used the Persuasive System Design (PSD) model as a framework for data extraction and analysis to examine how Internet-based CBT and IPT, as technology-based systems, influence the attitudes and behaviors of system users. PSD components described for the therapies were linked to reported outcomes using a cross-case comparison method and thematic synthesis.

**Results:**

We identified 19 Internet-based CBT programs in 59 documents. Of those, 71% (42/59) were of moderate to high quality. The PSD features surface credibility (competent “look and feel”), dialogue support (online program + in-person support), liking and similarity (esthetics and content appeal to adolescent users), the reduction and tunneling of therapeutic content (reducing online content into simple tasks, guiding users), and use of self-monitoring were present in therapies that resulted in improved therapy engagement, satisfaction, and adherence, as well as symptom and functional impairments.

**Conclusions:**

When incorporated into Internet-based CBT for adolescent depression, PSD features may improve adolescent adherence, satisfaction, and depression-related outcomes. Testing of these features using hypothesis-driven dismantling approaches is recommended to advance our understanding of how these features contribute to therapy effectiveness.

## Introduction

In their lifetimes, as many as 1 in every 9 adolescents will meet criteria for major depressive disorder (MDD) [1]. A comparable percentage of children and adolescents will also experience subthreshold depressive symptoms. MDD interferes with academic performance and school attendance, can have persistent impacts on daily functioning, and disrupts family and peer relationships [2-5]. For many adolescents, it is a recurrent and lifelong illness making access to timely assessment and treatment essential.

Cognitive behavioral therapy (CBT) and interpersonal psychotherapy (IPT) are recommended psychological therapies for adolescents with MDD [6]; yet, a considerable proportion of adolescents with MDD do not receive such services [7,8]. The reasons may include a lack of trained deliverers, inconvenient service times and locations, the social stigma associated with mental illness, discomfort discussing mental health problems, or a preference for self-help options [9,10].

While CBT and IPT have been traditionally delivered as face-to-face therapies, Internet-based (online) delivery of these therapies is proposed as a solution to access, availability, and uptake barriers. As technology-based treatments, Internet-based psychological therapies consist of (1) therapeutic content, (2) interaction between the user, their computer, and treatment material on the webpage, and (3) technological features embedded into the program (eg, multimedia, interactive treatment components). Recent systematic reviews of studies of Internet-based CBT and IPT for MDD have shown that Internet-based CBT and IPT therapies for clinical (adolescents diagnosed with MDD) and general (adolescents with subthreshold symptoms, adolescents considered at high risk for MDD) populations result in improvements in depressive symptoms and moderate to high satisfaction with the therapies [11-13]. Reviews have also highlighted variability across studies in terms of therapy attrition rates and the design features and functionalities of online delivery. This variability emphasizes the “black box” that remains for understanding how online CBT and IPT engage adolescent users, deliver therapeutic content, and lead to symptom improvements.

We report on a realist review that we conducted to examine the technological and program delivery features of Internet-based CBT and IPT for adolescent depression and to document their potential relation to treatment outcomes and program use. The realist approach provided a lens to explore two main questions: (1) Under what conditions are Internet-based CBT and IPT for adolescent depression being delivered? and (2) Within these conditions, what are the technological features of Internet-based CBT and IPT programs that may explain outcomes reported in studies?

## Methods

### Study Design

Our review used realist synthesis philosophy and principles as recommended by Pawson and Tilley [14] and is reported using Realist And Meta-narrative Evidence Synthesis: Evolving Standards (RAMESES) criteria [15]. Realist synthesis is an approach concerned with theory development and refinement of “how interventions work, for whom, and under what circumstances” [16]. From a realist lens, treatment effects are understood to be influenced by implementation context, and thus, Context-Mechanism-Outcome (C-M-O) configurations provide potential explanations about causal processes for treatment outcomes. Realist synthesis is also particularly useful when reviewing a complex and heterogeneous body of research (ie, diverse study designs, interventions used, outcomes measured) [14]. In this realist review, we examined the relationships between contextual factors for Internet-based CBT and IPT delivery (eg, conditions of use and the type of user, therapeutic content) and adolescent outcomes (eg, symptom reduction, satisfaction, therapy adherence), and the underlying mechanisms (eg, user behaviors, technological features/system design) that connect them.

### Theory Identification

Many realist reviews begin by identifying a theory or theories to develop a preliminary list of C-M-O configurations. The evidence identified in the review is then used to determine which C-M-O configurations are upheld when reviewing the evidence. In contrast, we identified potential theories for our review using an iterative process during project development—brainstorming within the review team and reviewing literature on human-technology interaction and studies of Internet-based psychological therapies for adolescent depression. Persuasive System Design (PSD) emerged from this process as a key framework for our C-M-O configurations. Being derived from both behavior change models and information technology systems models, PSD provided a comprehensive framework for exploring how systems influence the attitudes and behaviors of system users [17,18]. The framework is composed of four system features that relate to the persuasiveness of an Internet-based therapy—primary task support, dialogue support, system credibility support, and social support (Table 1). An Internet-based therapy can take on a persuasive role and use persuasive mechanisms in online delivery (persuasive system design) by (1) conveying symbolic (eg, text, data graphs, icons) and sensory content (eg, real-time video, virtual worlds, simulation) and (2) facilitating a social experience through the adoption of animate characteristics (ie, physical features, emotions, voice communication) and roles (eg, coach, pet, assistant, opponent), and using social dynamics (eg, greetings, apologies, taking turns) [17].

Using the PSD model, we then theorized what persuasive system features were likely to be linked to each outcome (ie, which features were mechanisms) reported for Internet-based CBT and IPT, and what delivery contexts were the most relevant in allowing that to happen. The end result of this discussion was a list of C-M-O configurations that would guide data analysis.

*Search for Relevant Literature* Realist methods call for a purposive and iterative approach. The search process began with a research librarian conducting a systematic search of academic databases in the psychology and health fields: Medline, CINAHL, Embase, PsycINFO for the period 2000-2016. Other search engines—Google and Beacon—and gray literature repositories (ACM Digital Library, OpenGrey, Canadian Agency for Drugs and Technologies in Health, IEEE Digital Library) were manually searched to identify gray literature such as government reports, community program evaluations, and conference proceedings for the same time period. Search terms were related to clinical area (ie, depression, mental health), modality (ie, online, Internet-based), and therapeutic approach (ie, CBT and IPT). The strategies for two searches are provided in Multimedia Appendix 1.

We also manually searched the table of contents in medical informatics journals (ie, Journal of Medical Internet Research, Internet Interventions, Journal of Cybertherapy and Rehabilitation, Journal of Telemedicine and Telecare). Snowball searching was also conducted (ie, reviewing eligible article reference lists) to identify relevant documents that may have been missed in the search process.

**Table 1:** The Persuasive Systems Design model.

Category	Persuasive feature	Definition
Primary task support	Reduction	Reduces complex behavior into simple tasks
	Tunneling	Guides a user through a process or experience
	Tailoring	Tailors the experience to the potential needs, interests, personality, or use context
	Personalization	Personalizes content (eg, allows you to customize the interface or populates your name)
	Self-monitoring	Keeps track of the user’s performance or status towards goal achievement
	Simulation	Provides simulations to enable the user to observe link between cause and effect
	Rehearsal	Provides a way for user to rehearse a skill or task
Dialogue support	Praise	Offers praise as a form of feedback
	Rewards	Rewards target behaviors
	Reminders	Reminds the user of their target behavior
	Suggestion	Offers fitting suggestions
	Similarity	Reminds the user of themselves in some meaningful way
	Liking	Is visually attractive for the user
	Social role	Adopts a social role
System credibility support	Trustworthiness	Provide information that is truthful, fair, and unbiased
	Expertise	Provides information showing knowledge, experience, and competence
	Surface credibility	Has a competent look and feel
	Real-world feel	Provides information of the actual people behind its content and services
	Authority	Refers to people in the role of authority
	Third-party endorsement	Provides endorsements from other sources
	Verifiability	Provides means to verify the accuracy of program via outside sources
Social support	Social learning	Can use the system to observe others performing tasks or behaviors
	Social comparison	Can use the system to compare their performance with the performance of others
	Normative influence	Leverages normative influence or peer pressure
	Social facilitation	User is able to discern via the system that others are performing the behavior along with them
	Cooperation	Leverages drive to cooperate to complete tasks or behaviors
	Competition	Leverages drive to compete against others in completing a task or action
	Recognition	Offers public recognition for an individual or group

### Literature Selection

Search results from academic databases were downloaded into Endnote (Thomson Reuters, Version 7.2) and then screened for eligibility by 2 trained raters (authors LW, AR). Inclusion criteria were as follows: (1) intervention studies (eg, clinical trials) were eligible for inclusion if they evaluated Internet-based CBT or IPT with adolescents with depression, (2) theoretical papers, mixed-methods and qualitative studies, and policy/implementation documents were eligible if they included a focus on how Internet-based CBT/IPT is proposed to work for adolescents with depression/depressive symptoms, and (3) documents that met Criteria 1 and 2 were eligible if they were published in English language. Any reviews (systematic, meta-analysis, etc) identified during our screening process were appraised to identify intervention studies and other potentially relevant documents.

During Stage 1 screening, the eligibility of a random subset (10 citations) was assessed independently by 2 team members (authors LW, AR), and interrater agreement was assessed within the “substantial” Kappa range (Cohen kappa=.74).

### Literature Appraisal

The evidence for each Internet-based therapy was assessed for relevance and rigor by consensus of 2 reviewers (authors LW, AR). These two reviewers also conducted co-coding and debriefing activities periodically during analysis.

Relevance was defined as the level of contribution to the review, and rigor was defined by the methodological quality of a study conducted on the Internet-based therapy (intervention, mixed-methods, and qualitative studies). Relevance was assessed by reviewing the details provided for an Internet-based therapy’s (1) context (eg, user, program features/design components), (2) mechanism(s): hypotheses as to how specific elements of the therapy worked, was proposed to work, or did not work, and (3) outcomes: reasons for therapy effect or lack of effect on specific adolescent outcomes. These details were obtained by reviewing documentation of usability evaluation, therapy/study protocols, and publications related to evaluations (eg, clinical intervention studies evaluating efficacy or effectiveness). Relevance was rated as low/none (no or little information), medium (some information), and high (well-described information).

The methodological quality of evidence (rigor) around each therapy was assessed, where possible, using the Mixed Methods Appraisal Tool (MMAT), an effective and practical quality assessment tool [19]. The tool includes sections for qualitative, mixed-methods, and quantitative studies. MMAT scores range from 0 (no criteria met) to 4 (all criteria met). Pawson describes the process of determining rigor as whether a “particular inference drawn by the authors has sufficient evidence to make a methodologically credible contribution to the test of a theory” [20]. Thus, we used the MMAT to assess the credibility of the reported findings based on the methodology described. Following MMAT guidelines, if publications were companion papers on the same data set, they were assessed as a set of publications.

### Data Extraction

NVivo software (QSR International; Version 11) was used to extract data. We extracted data for several aspects of the context of the Internet-based therapy: (1) user context (eg, urban/rural, age group, sociocultural composition, clinical severity), (2) usage context (eg, therapy objectives, adjunct versus stand-alone therapy), and (3) technology context (eg, synchronicity, use of multimedia, software and bandwidth requirements, mobile phone versus desktop). We also extracted information on therapy design (ie, conditions under which an adolescent completed the therapy: sequence, structure, timing; CBT/IPT features). In instances where the description of the therapeutic content was not available in the article, we included information from the article’s citations that described a therapy. We also extracted available information on PSD system features (primary task support, dialogue support, system credibility support, social support), therapy usage (eg, attrition, engagement), as well as clinical (eg, symptom reduction) and therapeutic (eg, therapeutic alliance) outcomes. Where available, information on full or partial C-M-O configurations was also extracted for individual therapies (ie, we sought data on particular therapies that could explain what Mechanism led to an Outcome, under which Contexts).

To promote consistency during data extraction, a coding guide with operational definitions for each code was used. PSD principles were used to guide the coding of mechanisms [14,18]. Two review team members (authors LW, AR) cross-referenced decisions regarding coding and extraction on a random subset of 10 articles and the remaining documents were coded by 1 reviewer (author LW).

### Analysis and Synthesis Process

We used a multistep approach to identify and organize information about what contexts of Internet-based CBT/IPT and persuasive system design attributes may contribute to adolescent outcomes. Our initial list of C-M-O configurations was revised based on consensus between team members. Drawing from qualitative synthesis methods, we selected an “index” therapy (CATCH-IT [21]) that was conceptually rich (ie, high methodological rigor, and most complete and robust descriptions) and could be used as a starting point for lines of argument synthesis [22] (ie, a form of grounded theorizing). Using a cross-case comparison method, we initially compared each therapy to the index therapy using the data matrix produced during data extraction. Similar and recurring concepts and themes were pulled together in order to corroborate or refute the proposed C-M-O configurations. This process involved identifying concepts from one therapy and recognizing the same concepts in another therapy, though they may not be expressed using identical words [23]. Proposed C-M-O configurations were analyzed at different levels of abstraction (ie, within and across therapies) to determine the most robust and plausible explanations of how in a context, with a mechanism, outcomes could be generated.

As a final step we compiled a framework matrix for each C-M-O configuration in NVivo in order to map “demi-regularities”, semi-predictable patterns of therapy outcomes [16]. This allowed the review team to examine which therapies provided what evidence to support each C-M-O configuration. Conference calls with team members were used to discuss, amend, or confirm identified patterns. In instances where a C-M-O configuration was judged to not fully explain the demi-regularities, we returned to the data to refine the configuration.

## Results

Figure 1 presents a flow diagram outlining the document search and appraisal process. A total of 15,760 unique and potentially eligible documents were reviewed for inclusion in this review. Of these, 59 documents were deemed eligible for inclusion: published studies (n=45), gray literature documents (n=8), and clinical trial protocols (n=6). These documents were published between 2006 and 2016, and they detailed 19 unique Internet-based psychological therapies.

### Characteristics of Internet-based Therapies for Adolescent Depression

#### Structure and Delivery Features

An overview of the structure and delivery features of the 19 Internet-based therapies is provided in Table 2. The majority of the therapies (14/19, 79%) were adaptations from manualized (paper-based) programs, Internet-based therapies designed for adults, or other online programs. Six therapies were designed to concurrently treat depressive and anxiety disorders (Cope2Thrive, Chilled Plus, MoodGym, Mood Mechanic Course, ThisWayUp, Feeling Better), and two therapies were designed to also address problems with alcohol use (DEAL, iTread). Two therapies had significantly larger bodies of associated research than the others—of the reviewed documents, 19% (11/59) were related to the MoodGym program and 15% (9/59) were related to the CATCH-IT program.

Most therapies (14/19, 74%) were designed to support contact with a health care or teaching professional. However, the nature of this contact varied significantly across therapies ranging from a 10-minute, initial face-to-face motivational interview (CATCH-IT) to a fully synchronous, online chatroom moderated by a trained coach over multiple weeks (Master Your Mood). Three therapies included the option of synchronous computer-mediated communication (ie, chat, text messaging); most therapies relied on email-based communication. ChilledPlus, CATCH-IT, and CURB involved the adolescent’s primary health care provider and adjunct education for parents as part of the therapy. For brief content descriptions of each Internet-based therapy, see Multimedia Appendix 2.

**Table 2:** Reported structure and delivery characteristics of Internet-based psychological therapies for adolescent MDD.

Program (country)	Participants			Program details				
	Target age (years)	Testing context^a^		Parent involvement	Time commitment	Contact		Adapted from
						Before program	During program	
Blues Blaster (USA) [24]	11-15	P		No	Total: 60-90 minutes over 1 wk 6 modules (10-15 minutes per module); 1 module/day	None	None	Face-to-face Coping With Depression
CATCH-IT (USA) [21,25-32]	14-21	P		Yes	Total: 660-840 minutes over 7-8 wks 11-14 modules (60 minutes per module); 1-2 modules/wk	In-person^b^	Phone	
Chilled Plus (AUS) [33,34]	12-17	T		Yes	Total: 600 minutes over 8 weeks 8 modules (60 minute per module + 30 minute phone calls); 1 module/wk	In-person	Email	Face-to-face Chilled
Cope2thrive (USA) [35,36]	13-18	P, T		No	Total: 350 minutes over 7 weeks 7 sessions (50 minutes per session); 1 session/wk	None	Email/ Phone	Face-to-face COPE group
CURB (USA) [37]	13-17	P		Yes	Total: 660-840 minutes over 7-8 weeks 11-14 modules (60 minutes per module); 1-2 modules/wk	In-person	Phone	CATCH-IT
DEAL (AUS) [38-41]	18-25	T		No	Total: 240 minutes over 4 wks 4 sessions (60 minutes per session)	None	Email	Computerized SHADE
DWD (CAN) [42,43]	13-18	P, T		No	Total: unspecified 8 sections	None	None	Manualized DWD
Feeling Better (CAN) [44,45]	16-30	T		No	Total: 120-200 minutes over 6-10 wks	None	Email/ Phone	Telehealth Family Help
iRFCBT (UK) [46]	15-22	P		No	Total: 360 minutes over 6-12 wks 6 modules (60 minutes per module); 1 module/1-2 wks	None	Email	Internet MindReSolve
iTreAD (AUS) [47]	18-30	T		No	Total: minimum 240 minutes + social networking over 12 months 4 sessions (60 minutes per module)	None	Email/ Online chat	Includes DEAL as component
Master Your Mood (NZ) [48-52]	16-25	T		No	Total: 720 minutes over 8 wks 8 modules (90 minutes per modules); 1 module/wk	None	Online chat	Face-to face group Grip op je dip
MAYA (Chile) [53-55]	12-18	T		No	Total: <20 minutes over <1 wk 1 session	None	None	
Mood Mechanic Course (AUS) [56,57]	18-25	T		No	Total: 1924 minutes over 8 wks 5 lessons	None	Email/ phone/ text	Internet UniWellbeing
MoodGym (AUS) [58-68]	12-17	P, T		No	Total: 150-300 minutes over 2-3 wks 5 modules (30-60 minutes per module); 1-2 modules/wk	None^c^	None	
MoodHelper (USA) [69]	18-24	T		No	Total: unspecified 4 sessions	None	None	Internet for adults ODIN
OIPE (USA) [70]	12-17	T		No	Total: unspecified number of minutes over 12 wks 8 modules	In-person	Text	
Rebound (AUS) [71,72]	15-24	RP		No	Total: unspecified number of minutes over 12 wks User can select from 56 sessions (20 minutes per session)	None	Social network moderation	Internet Horysons for youth psychosis
SPARX (AUS) [73-79]	13-18	P, T		No	Total: 210 minutes 7 modules (30 minutes per module)	None^d^	Phone	CD-ROM SPARX
Thiswayup (AUS) [80,81]	12-16	P		No	Total: 228-263 minutes over 7 wks 7 modules (per module: 15-20 minutes online + 17.5 minute discussion); 1 module/wk	In-person	In-person	Face-to-face CLIMATE Schools

^a^Testing context refers to the type of population who received the therapy: P=prevention (ie, recruited participants with subthreshold depression), T=treatment (ie, inclusion criteria stipulated that participant meet threshold for depressive symptomology, risk, or diagnosis), RP=relapse prevention (ie, required participant have had a previous depressive episode)

^b^Contact with primary care provider was either motivational interview or brief advice.

^c^MoodGym was tested in different implementation contexts; some included no in-person contact and some with in-person contact.

^d^SPARX was tested in different implementation contexts; some included no in-person contact and some with in-person contact.

**Figure 2:**
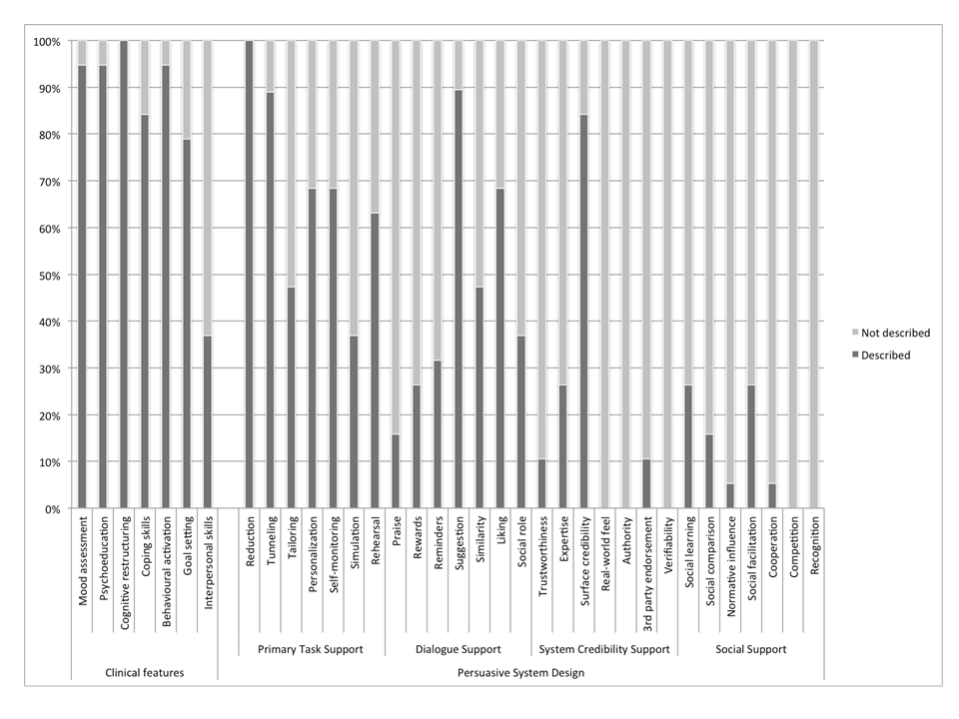
Reported therapeutic and persuasive system design features in evaluated Internet-based psychological therapies for adolescent MDD.

#### Psychological Therapy Approaches

All 19 therapies were based on principles of CBT and used essential “ingredients” described in clinical practice guidelines (eg, [82,83]). Fidelity to CBT was high for 14 therapies that described 6/6 core ingredients (Figure 2). Fidelity to CBT was modest for 4 therapies that described 4 or 5 out of 6 core ingredients, and low for 1 therapy (MAYA) that included 3/6 core ingredients. Seven therapies (CATCH-IT, CURB, DEAL, iRFCBT, Rebound, MAYA, Chilled Plus) also included an interpersonal therapeutic orientation (eg, social support, interpersonal conflict resolution) consistent with principles of IPT [84].

#### Persuasive System Design Features

The PSD features that were present in the therapies are also presented in Figure 2. Primary task support and dialogue support were found to be the most widely represented. All of the therapies included some reference to *reduction*, and a majority referenced some form of *tunneling* and *suggestions* (17/19 therapies). Aspects of system credibility support were not well represented in the documentation we reviewed for the therapies with the exception of *surface credibility* (16/19 therapies). Persuasive design features that leverage social support to motivate users were rarely, if ever, reported features.

### Level of Contribution and Methodological Quality

Details of the level of contribution and quality assessments are provided in Table 3. Based on the level of contribution assessment, three therapies—MoodGym, SPARX, and CATCH-IT—were rated as having high contributions to all three dimensions relevant to the review questions: contexts, mechanisms, and outcomes. Twelve other therapies provided medium to high contributions to at least one of the three dimensions. Contributions from four therapies were low. Across the therapies, descriptions of mechanisms (how technology components were expected to function in relation to outcomes) and context were less developed than descriptions of outcomes. The methodological quality of studies associated with each therapy varied considerably. Half (28/57) of the documents were research studies eligible for MMAT ratings—15 randomized controlled trials, 3 nonrandomized trials, 3 quantitative descriptive studies, 4 mixed-method studies, and 3 qualitative studies. All studies met at least 2 of four MMAT criteria. Studies with lower ratings did not provide a clear description of the randomization process, had higher than 20% study dropout rates, or used nonvalidated measurement tools. Follow-up beyond 6 months was conducted only for the CATCH-IT program suggesting limited knowledge about post-program experiences of adolescents.

**Table 3:** The availability and contribution of evidence related to Internet-based psychological therapies.

Therapy	Level of contribution^a^			Documentation available for review			Associated MMAT scores^a^
	Context	Mechanism	Outcome	Usability	Protocol	Efficacy/Effectiveness	
CATCH-IT	High	High	High	[27,29,30]	[26]	[21,25,28]	4 [21], 4 [25], 3 [29], 3 [30]
MoodGym	High	High	High	None	None	[58-68]	3 [58], 3 [59], 2 [60], 3 [61], 2 [62], 4 [63-64], 4 [65], 2 [66], 3 [67] [68]
SPARX	High	High	High	[74,75,78]	[79]	[73,77]	4 [73], 3 [74], 3 [75], 4 [77], 3 [78]
Blues Blaster	High	High	Medium	None	None	[24]	3 [24]
DEAL	High	Medium	High	[38]	[40]	[39,41]	3 [38], 3 [39,41]
Master Your Mood	Medium	High	High	None	[51]	[48,49]	2 [48], 2 [49]
MoodHelper	Low	High	Medium	None	None	[69]	3 [69]
Feeling Better	Medium	Medium	Low	[44,45]	None	None	2 [44], 2 [45]
Thiswayup	Medium	Low	Medium	None	None	[80,81]	2 [80], 4 [81]
Maya	Low	Medium	Medium	[53,54]	None	[55]	2 [53-55]
OIPE	Low	Low	Medium	None	None	[70]	2 [70]
Mood Mechanic Course	Low	Low	Medium	None	None	[56]	3 [56]
Rebound	Medium	Medium	Low	[71]	None	[72]	3 [71], 4 [72]
Cope2thrive	Medium	Low	Medium	None	None	[35]	3 [35]
CURB	Medium	Low	None	[37]	None	None	N/A
iRFCBT	Low	Low	None	None	[46]	Ongoing trial	N/A
iTreAD	Low	Low	None	None	[47]	Ongoing trial	N/A
Chilled Plus	Low	Low	None	None	None	Ongoing trial	N/A
DWD	Low	Low	None	None	None	Open Access	N/A

^a^Following published guidelines for MMAT scoring, in instances where multiple documents reported on the same data set, a single MMAT score was calculated.

### Context-Mechanism-Outcome Configurations

Of the candidate C-M-O configurations initially put forward using the PSD model, five configurations were substantively supported by available evidence (Table 4). We present the configurations with key examples of the contexts, mechanisms, and outcomes from the documents reviewed.

#### C-M-O Configuration 1

In this review, we found that *dialogue support* provided as a PSD feature by a therapy required real-time, in-person guidance to optimize therapy adherence. Five therapies contributed to this C-M-O configuration, which were connected to studies with a moderate mean MMAT score of 2.94 (SD 0.77).

Real-time guidance involved adolescents completing the Internet-based therapy with an individual (doctor, teacher, therapist) in a setting available to support them (or supervise them) while they completed the activities. Results from studies suggested that completion rates increased if the therapy was delivered with real-time guidance in contexts such as schools or connected with primary/secondary care versus having the adolescent complete the therapy on their own (self-guided) [21,58,80]. Even therapies that included more computer-generated or automated dialogue opportunities (ie, reminders, praise, automated suggestions) were able to optimize adherence only with real-time, in-person contact [27,73,80].

Studies of MoodGym have compared in-person guidance to self-guidance in a setting of their choice (eg, home) with synchronous support from the virtual guide (eg, avatar). One study found that there was a 10-fold difference between the approaches (favoring the in-person guidance) in terms of the number of online exercises adolescents completed [58]. The authors of the study stated that the comparison highlighted “the success of the monitored setting in increasing compliance” [58] Sethi et al have suggested that, “treating youth depression with a combination of face-to-face and online therapy is ideal...if the technology incorporates ways to interactively learn and practice at their own pace” [68]. Reasons for nonuse reported by adolescents in one MoodGym study showed that almost a third of adolescents indicated they stopped using the therapy because they, “felt the need to talk to someone, rather than doing this program.” [65].

**Table 4:** Summary of the C-M-O configurations substantively supported by evidence.

C-M-O configuration		C, M, O	Supporting programs
1. Computer-mediated dialogue required real-time support and monitoring to optimize therapy adherence.		C: real-time support M: dialogue support features O: adherence	CATCH-IT, MAYA, MoodGym, SPARX, ThisWayUp
2. Therapies with surface credibility led to engagement and satisfaction with the therapy.		C: user interface M: credibility support O: engagement and satisfaction	Blues Blaster, CATCH-IT, Feeling Better, Master Your Mood, MAYA, SPARX
3. Therapies that included liking and similarity features led to engagement and satisfaction with the therapy.		C: user interface M: liking and similarity O: engagement and satisfaction	Blues Blaster, CATCH-IT, CURB, DEAL, Feeling Better, MoodGym, MAYA, SPARX
4. Reduction and tunneling of therapy content were necessary for adolescents to complete more of the therapy.		C: user interface M: reduction and tunneling O: adherence	Blues Blaster, CATCH-IT, CURB, DEAL, Feeling Better, Master Your Mood, MAYA, MoodGym, SPARX
5. Self-monitoring was a key PSD component for facilitating symptom improvements among adolescent users with a MDD diagnosis or functional impairments.		C: users with a MDD diagnosis and/or functional impairments M: self-monitoring O: clinical outcomes	Blues Blaster, CATCH-IT, DEAL, Feeling Better, Master Your Mood, Mood Helper

#### C-M-O Configuration 2

In this review, we found that adolescents assess the *surface credibility* of an Internet-based therapy, and that this assessment impacts engagement and satisfaction with the therapy. Therapies with surface credibility have resulted in higher engagement and satisfaction. Others have argued that surface credibility is critical “as negative perceptions of the interface usability could influence the program’s eventual effectiveness...acceptability... [and] long-term clinical value” [45]. Six therapies contributed to this C-M-O configuration, which were connected to studies with a high mean MMAT score of 3.10 (SD 0.79).

Potential therapy users quickly assess credibility [45] and look for programs that have good face validity and are appealing [45]. Adolescents have been found to be highly satisfied with programs that presented therapeutic key concepts in engaging ways (eg, video, animation, interactive exercises) [24] and have reported that multimedia resources make programs “easier to use” and “helped in learning the material” [24]. Features perceived as unacceptable to adolescents include games that are not engaging or interactive, sections that are difficult to navigate, and video streaming that lags [53,55]. Adolescents are significantly more likely to complete a therapy designed in response to adolescent consumer preferences [27]. In a study of Master Your Mood, 50% of participants quit before completing half of the program and attributed this in part, to the fact that they “did not feel motivated by the materials provided” [49].

#### C-M-O Configuration 3

Therapies that incorporated the PSD elements of *liking* (ie, visual appeal) and *similarity* (ie, a way for adolescents to recognize themselves in the program) were found to lead to increased adolescent engagement and satisfaction. Eight therapies contributed to this C-M-O configuration; supporting primary studies had a high mean MMAT score of 3.16 (SD 0.76).

Across programs, liking related to the program’s appearance. The issue of color palette was consistently identified during usability testing [38,44]. For example, the culturally adapted version of CATCH-IT for African-American and Latino adolescents (CURB) was designed to “avoid the appearance of a ‘school-like’ experience” [36] and later redesigned after adolescents reported that the “initial color design appeared too ‘boring’” [37].

Feedback on the experience of the online video game MAYA, which had low satisfaction and engagement, suggested that, “it would be desirable that [the game] portrayed a social context more similar to [the participant’s] reality” [55]. A central theme for several programs has been similarity. Programs have found that cultural relevance, the use of personal characters and language for adolescents to relate to, as well as tailoring content based on user choices, has led to increased adolescent engagement and satisfaction [38,44,75]. As one participant in the Feeling Better program indicated, “it [tailored content] made me more interested in it, like rather than just kind of skipping through it” [44]. In exploring nonsignificant or mixed-results in several studies of MoodGym among adolescent populations, Sethi et al speculated that because the program was originally developed for adults, it “may need to be tailored to specific adolescents themes and examples to encourage engagement” [62].

#### C-M-O Configuration 4

The use of *reduction* and *tunneling* of therapeutic content has been found as necessary for adolescents to achieve therapy adherence. In this way, therapies that initiate young people into doing a series of therapeutic actions through incremental steps (small doses) rather than a one-time consolidated effort may provide a more persuasive experience that leads to improved outcomes. This C-M-O configuration is supported by evidence from 9 therapies and studies with an average MMAT score of 2.81 (SD 0.66) suggesting overall moderate quality evidence.

Reducing the amount of text, improving navigational instructions, and reducing the length of therapy modules to improve ease of use were associated with higher adherence (eg, [30,32,39]). Heterogeneity of therapies made it challenging to disentangle under what contexts tunneling and reduction worked more/less persuasively in improving adherence. Large variations in program design: (1) time commitment per module (range 20-1924 minutes), (2) number of modules (range 4-14), (3) attrition/dropout out measures used, and (4) differences in self-paced versus locked content (“unlocked” until the completion of certain therapy tasks/activities) were problematic when attempting to compare these persuasive design features across therapies. Moreover, many of the therapies targeted a wide range of ages without providing discussions on how developmental level or cognitive ability were considered in terms of reduction and tunneling strategies. Less than half (8/19, 42%) of the therapies targeted young people with a spread of 7 years or more (eg, Feeling Better was designed for young adults 16-30 [14-year range] and Master Your Mood was designed for 16-25 year olds [9-year spread]).

#### C-M-O Configuration 5

A defining feature of the therapies that were successful in treating adolescents with severe symptomology at baseline was the use of *self-monitoring* as a PSD feature. This C-M-O configuration was supported by evidence from 6 therapies with high-quality evidence (mean MMAT score, mean 3.09, SD 0.54).

Increasing self-awareness of emotions is an important clinical program feature for treating depression as it prepares individuals for changing their cognitions, beliefs, and schemas [82,83]. Studies contributing to this C-M-O configuration were rated as having modest to high fidelity to CBT (5 or 6 out of 6 clinical features; see Figure 2). Thus, opportunities for self-monitoring likely played a role both as an active therapeutic ingredient but also as a PSD element. Two studies provide useful case examples of the impact of self-monitoring.

Mood Helper was one of the only pure stand-alone Internet-based therapies in our review [69]. Mood Helper comprises multiple opportunities for self-monitoring. Users (1) complete a series of brief auto-scored depression scales, (2) review their graphically displayed depression scores over time, (3) receive automated reminders to return to the website every few days, (4) are given access to a personal diary space, and (5) are guided in creating self-contracts to define goals and remind the user of progress towards them. A small but significant effect size favoring Mood Helper compared to the treatment as usual control condition was found, and researchers noted that participants using the website more intensely had higher average baseline depression scores [69]. This was especially significant since researchers found an unexpected dose-effect response where fewer minutes of the website usage were associated with greater depression symptom reduction. A post-hoc analysis showed that participants who improved more rapidly found the website less necessary and they discontinued use, “whereas those with more persistent depression may have continued for longer periods in the hopes of obtaining relief” [69]. This finding suggests that tailoring self-monitoring around level of depressive symptoms maybe be warranted as it might facilitate and likely trigger increased engagement in practicing skills and in turn, decreasing symptoms.

The second program, Blues Blaster, was designed primarily to teach adolescents how to monitor their mood and to do so using engaging methods [24]. As adolescents progress through each of the six modules they are “encouraged to track their mood ratings and fun activities which are depicted together on a graph to help illustrate the relationship between them” [24]. Adolescents also track personal “mood triggers” to help them plan ahead. Mean responses on the 6-point satisfaction items, “I liked seeing the graph of my mood and activities” (mean 4.52, SD 1.27) and “the mood and activities tracking form was easy to fill out” (mean 4.58, SD 1.15) showed strong support for the self-monitoring components of the program. Results of a randomized controlled trial demonstrated greater improvement for the Blues Blaster condition in depression levels, negative thoughts, behavioral activation, knowledge, self-efficacy, and school functioning compared to the information-only control condition [24]. A significant correlation was also found between total modules completed and the depression measure posttest change scores.

## Discussion

### Principal Considerations

Advances in technology have allowed for health care programs to connect users to treatments in dynamic ways. The dramatic growth of technologies designed to persuade and motivate represents a significant shift in focus toward end-user computing in health behavior change therapies [18]. Internet-based therapies for adolescent depression are one group of therapies that have incorporated persuasive design strategies to influence therapy outcomes. However, to date, such programs have not been reviewed and evaluated with this perspective. Not accounting for human-technology interaction in therapy design and evaluation limits our understanding of how Internet-based therapies work. In our view, this review of Internet-based CBT and IPT for adolescent MDD highlights the need to purposefully consider PSD features early in the program design process and to further develop PSD theory to help explain therapy outcomes. This review is also an important step in understanding how PSD features of Internet-based therapies may work based on existing literature. The C-M-O configurations generated by this review represent important hypotheses. The testing of specific program mechanisms (ie, PSD features, provider involvement) through hypothesis-driven, dismantling approaches is now necessary to advance the understanding of the effect of Internet-based therapies on users.

A key argument in favor of developing and offering Internet-based psychological therapies to young people is increased access [57] to effective mental health care. Therapies in our review were either stand-alone therapies meant to increase options for young people with depression [69] or used in combination with existing health care [25] or school-based [58] services. Therapies that relied on existing services reported better engagement with the program and completion rates, although across all studies, attrition was a reported issue. Does availability matter if young people do not complete the program as intended? At this point in time, this question is hard to answer. It is necessary for future studies to examine whether young people stop using stand-alone therapies because of symptom improvement, changes in motivation, or because of the program features. Qualitative exploration of the type of support provided and required during adolescent use of programs used in combination with existing services would also provide valuable information on necessary “program ingredients.” While adherence to Internet-based programs has traditionally been low [85], the use of PSD principles, or in-person support from existing services at critical program timepoints (eg, when dropout has been shown to occur, when therapeutic content is strenuous for the user) may address program engagement and adherence.

The findings from our review, in terms of surface credibility and liking, suggest that adolescents’ visual experiences lead to esthetic and credibility judgments [86-88] and should also be taken into account. Bennett and Glasgow have argued that the most critical design gap in the current generation of Internet-based programs is the underuse of Web 2.0 features (eg, social media, user-generated content, collaborative consumption) [89]. In our review, many therapies were adapted from face-to-face or manualized programs. This may have resulted in a “replication” of components that were originally designed for face-to-face interactions rather than fully optimizing Web 2.0 features available for delivery.

Fogg has argued that persuasive systems will work only if the user has sufficient motivation and the user’s ability is being adequately triggered to perform the new behavior [17]. High rates of noncompletion of Internet-based psychological therapies for adolescent MDD suggest the need for therapies to address motivation in the context of depression/depressive symptoms (eg, if a depressed adolescent is not motivated to log into a stand-alone program, what program supports are needed for behavioral activation?). That the use of specific PSD features—reduction, tunneling, self-monitoring—led to increased adolescent engagement and satisfaction, and improvement in depressive symptoms and functioning, suggests the cognitive load of therapies may also need to be addressed. The advantage of primary task supports—tunneling, reduction, and self-monitoring—is that these PSD features can increase positive affect (ie, “I can do this”) [90,91]. Pairing primary task supports with other persuasive strategies such as self-monitoring, reminders, simulation-rehearsal, and suggestion-reward can be especially effective together [92,93]. Of the studies in this review, despite high attrition, most reported a positive clinical outcome associated with Internet-based program use, suggesting that we do not yet have a clear understanding of therapy “dosage” for adolescents and how “persuasive” a therapy needs to be (eg, the persuasive experience) to ensure optimal outcomes for adolescents. Future research is needed to strengthen our understanding of the relationship between treatment adherence and symptom change. Persuasive system design may be one testable model to facilitate knowledge generation. The studies in this review highlight that the relationship between engagement in therapy activities, adherence through to treatment completion, and treatment satisfaction are complexly related to clinical outcomes and require theoretical and empirical investigation.

Future studies should operationalize each PSD feature, hypothesize its intended effect, and measure its use and effect. As technology and methods of human-technology interaction evolve, this documentation and evidence will provide a valuable roadmap for the depression treatment field. Studies that use a factorial design or fractional factorial design would move the field forward by providing an opportunity to compare intervention groups that include multiple, and different combinations of, persuasive design functionality. For example, there could be conditions within both study arms in which some groups receive tailored feedback and others do not, some include a social networking forum and others do not, and some provide reminders and others do not. In this way, the impact of PSD features can be isolated. It might also be useful to develop hybrid designs that include both standard “randomization” as well as “preference” arms, in order to determine which groups of adolescents might be more attracted to certain PSD features.

The exploration of target population characteristics is also needed to determine how motivational (eg, readiness for change, self-regulatory skills), developmental/age-related, sociocultural, technical competency and modality features (synchronous, ambient, etc), and depression severity differentially interact with program mechanisms and impact adolescent outcomes. For example, research has shown gender differences in the perceived persuasiveness of numerous health intervention components, with females being more receptive to most persuasive behavior change strategies [94]. Under this approach, studies can identify mediators of therapy effect and examine both mediators and moderators as a means to identify program mechanisms (how) and for whom the therapy works best (who), respectively.

### Review Strengths and Challenges

This review is the first to use a realist framework for studying Internet-based psychological therapies for adolescent depression. This framework allowed us to consider studies and theories together to understand how the therapies worked. We included numerous therapies identified in the gray literature, which allowed for a comprehensive appraisal of the current evidence base and reduced the risk of publication and selection bias. Previous reviews of Internet-based therapies for this population have focused only on empirical literature, and therefore, have provided limited insight into the complex causal pathways that may underpin therapy effects. Including multiple research designs, while challenging from a data integration standpoint, enabled the analysis to benefit from the strengths of each approach and corroborate findings across divergent contexts and theoretical orientations. From a realist perspective, this diversity has huge explanatory value and can help uncover contexts and conditions not typically captured in meta-analytic or traditional systematic reviews. In addition to offering a more thorough assessment of Internet-based therapies, this review supersedes existing reviews by including substantially more therapies and documenting the body of work around each one. A further strength of the review is the use of gold standard review methods (notably, duplication of screening, quality assessment, and consensus-building with research team members).

The greatest challenge in applying the PSD model is that no explicit heuristics have been defined for it yet, and so nuances between different PSD features are still being mapped [95]. The model would gain more strength from explicitly defined scales and instructions for evaluating the implementation of each PSD principle. A further challenge in exploring these therapies from a sociotechnological frame is that studies largely employed traditional clinical reporting methods with very little attention paid to describing the informatics architecture and the *expected* role of technology functionalities. In this regard, usability studies included in our review provided much needed information. Because we reviewed the literature and evidence base around these therapies, as opposed to conducting a workflow analysis of each therapy, our appraisal was limited to what authors reported, which may or may not be a full reflection of the therapy’s capabilities and design.

Another challenge in our review was the lack of information on the nature of adolescent’s interaction with PSD features. For example, current descriptions of dialogue support provided in Internet-based therapies are lacking. While authors described using email, reminders, and options for peer engagement, there was little detail about actual engagement with these features (eg, How often were reminder emails triggered?, How many adolescents elected to publish journal entries to their peers?, How many adolescents spontaneously emailed their assigned coach and how often?). In terms of peer-based dialogue support, some have argued that there is not yet very strong evidence for what type of peer-based social support therapies ought to provide [96].

### Conclusions

Results from our review suggest there is room for improvement in both designing and implementing Internet-based therapies for adolescent depression and in elucidating how persuasive mechanisms are designed and ultimately function. We offer that many of the assumptions that implicitly shape Internet-based therapy development and delivery—adolescents are highly competent technology users, adolescents want to complete programs on their own, the more persuasive design components the better, or that compliance will result in improved outcomes—are vastly under-acknowledged and are based on pervasive assumptions about adolescents, what they prefer, and what they need. Improved engagement of adolescents with MDD in the design and development of future therapies is crucial if we hope to provide effective Internet-based therapies for this population.

## Appendix

Multimedia Appendix 1 Review search strategies.

Multimedia Appendix 2 Brief description of Internet-based psychological therapies for adolescent depression.

## Abbreviations

CBT: cognitive behavioral therapy
C-M-O: context-mechanism-outcome
IPT: Interpersonal Psychotherapy
MDD: major depressive disorder
MMAT: mixed method appraisal tool
PSD: persuasive system design
