# Supplementary material for: How do eHealth Programs for Adolescents With Depression Work? A Realist Review of Persuasive System Design Components in Internet-Based Psychological Therapies
Source: J Med Internet Res. 2017 Aug 9;19(8):e266. doi: 10.2196/jmir.7573 (PMC5569246; doi:10.2196/jmir.7573)
Supplement: Multimedia Appendix 1 [file jmir_v19i8e266_app1.pdf]

Review search strategies from (a) Google search and (b) Ovid Medline.

(a) Google search date: Feb 12, 2015 and updated Aug 3, 2016

1. online AND depression AND teens OR adolescents OR youth
2. internet-based ORweb-app OR web-based AND teens AND depression
3. depression AND internet therapy
4. Ehealth AND teen depression
5. mental health AND online AND youth OR teens
6. Internet AND mental health AND depression
7. youth AND depression AND online treatment
8. teens OR adolescent OR young adult AND internet AND cognitive behavior therapy AND depression

(b) Ovid Medline search date parameters: Feb 12, 2015 and updated Aug 3, 2016

1. Cellular Phone/
2. Computers/
3. Electronic Mail/
4. Internet/
5. Text Messaging/
6. Therapy, Computer-Assisted/
7. (android* or apps or cell* phone* or cellphone* or computer* or digital* or e health* or ehealth* or e mail* or email* or electronic mail* or internet* or iPad* or iPhone* or iPod* or m health* or mhealth* or mobile* or online* or personal digital assistant* or short messag* or smart phone* or smartphone* or technolog* or text messag* or virtual* or web*).tw.
8. or/1-7 [Combined MeSH and keywords for internet based technologies]
9. Behavior Therapy/
10. exp Cognitive Therapy/
11. Disease Management/
12. exp Directive Counseling/
13. Persuasive Communication/
14. Problem Solving/
15. Psychotherapy/
16. Self Care/
17. acceptance adj commitment therap*.tw.
18. (behavio* adj (activation or condition* or modif* or therap*)).tw.
19. CBT.tw.
20. cognitive therap*.tw.
21. directive counsel*.tw.
22. interpersonal therap*.tw.
23. mental health program*.tw.
24. mindfulness.tw.
25. motivational interview*.tw.
26. problem solving*.tw.

27. psychotherap*.tw.
28. (selfcar* or self car* or selfhelp* or self help* or selfmanage* or self manage* or selfmonitor* or self monitor*).tw.
29. telemental health*.tw.
30. or/9-29 [Combined MeSH and keywords for CBT]
31. Anxiety/
32. Anxiety Disorders/
33. Depression/
34. Depressive Disorder/
35. Mental Disorders/
36. Mood Disorders/
37. (anxi* or behavio?r* disorder* or behavio?r* problem* or depress* or mental disorder* or mental health* or psychiatric illness* or psychiatric disease* or psychiatric disorder*).tw.
38. or/31-37 [Combined MeSH and keywords for anxiety]
39. and/8,30,38 [Combined results for internet based technologies, CBT and anxiety searches]
