# Supplementary material for: How do eHealth Programs for Adolescents With Depression Work? A Realist Review of Persuasive System Design Components in Internet-Based Psychological Therapies
Source: J Med Internet Res. 2017 Aug 9;19(8):e266. doi: 10.2196/jmir.7573 (PMC5569246; doi:10.2196/jmir.7573)
Supplement: Multimedia Appendix 2 [file jmir_v19i8e266_app2.pdf]

Brief description of Internet-based psychological therapies for adolescent depression.

Program	Description
Blues Blaster	Users are given a login. On the website the user progresses through lessons that each involve: (1) a video to introduce the lesson goal along with a graphic illustration of the goal; (2) a Flash animation activity to teach the lesson's teaching points; (3) a Flash animation activity to reinforce the lesson's teaching points; (4) a comic strip depicting characters role playing the key concept presented in the lesson; (5) a video testimonial speaking to the lessons topic; (6) the lessons 'key', or summary statement; (7) quiz questions on the lessons content; and (8) a summary video providing positive reinforcement and encouragement.
CATCH-IT	Program involves an initial motivational interview with a family physician during clinic visit. On the website, the user progresses chronologically through modules that involve: learning goals, review, core concept explanation, adolescent stories to illustrate the lessons, skill building exercises, a summary, feedback on the experience and an Internet-based reward. Some studies included follow-up with a physician at different intervals (4-6 weeks, 3 times a year, 1 coaching call per month, 3 'check in' calls). Parents complete a complementary online 4- session program.
Chilled Plus	The user visits their primary health care provider. Both the user and the health care provider are interviewed before the user is given access to program. The cost of the program is a $100 bond, with $50 refunded after post-therapy follow-up and $50 refunded after 3-month follow-up. On the website, the user progresses chronologically through modules. Each module includes a 30-minute phone call with a therapist. The therapist is also in contact with the primary care provider and parents via phone throughout the program.
Cope2thrive	On the website the user views a narrated slideshow that illustrates key points in each of the seven 20- to 30-minute sessions. The user is encouraged to try out the principles presented and write down their own thoughts and reactions using the interactive component. Upon completion of the online program, the adolescent can print out a Certificate of Completion.
CURB	The program is a cultural adoption of the CATCH-IT programs and involves an initial motivational interview from family physician during clinic visit. On the website, each module includes learning goals, review, core concept explanation, stories to illustrate the lessons, skill building exercises, a summary, feedback on the experience and an Internet-based reward. The user revisits the family physician at 3 months and receives coaching calls at weeks 1, 2, 3 and 7 weeks. Parents complete a 3

	module complementary program with videos. The parent program is also available in Spanish.
DEAL	The DEAL program is designed to treat co-occurring depression and problematic alcohol use. On the site the user selects a pseudonym username that serves as their login. They are given 10 weeks of access to the 4-week program and complete sessions in chronological order at their own pace. The website tracks their progress through each weekly module and sends automated reminder emails after periods of inactivity.
DWD	An interactive website where the user can freely access and interact with short animations, listen to audio stories, click to access additional information on topics, hyperlink to other resources. Chronological order is implied but not required.
Feeling Better	On the website the user progresses chronologically though modules. There are two versions of the program (male and female). A standardized assessment of symptoms of distress is built into the start and end of the core program modules to monitor symptom change and help the user choose customized streams of program content specific to their emotional distress. Each module includes a phone coaching session.
iRFCBT	The user works through the content on a password protected website content and send responses via the program (integrated email) to the coach. The coach provides feedback on these responses within 2 working days, in particular, any positive steps made and encouraging users to sustain these as well as pointing out areas to focus on over the next module. Each module involves mood diaries, online experiential exercises using audio-recordings, video vignettes. Reminder emails are sent after 2 weeks if the user did not complete the module.
iTreAD	A web-based and mobile application that integrates a website and moderated mental health support communities. A closed (restricted access) Breathing Space social networking space is moderated by Australian research clinicians associated with the project. The user is able to interact with peers during the hours of 9 am to 9 pm to post their thoughts, messages of support, update their status, tell their stories, share self-assessment results, and report on progress related to their mood and binge drinking (successes and challenges). The user works through psychoeducation and skill building activities on the website. Notifications of new posts, reminders are sent via email addresses and the mobile application.
Master Your Mood	The user is assigned to a small chat group (6-8 max) with defined live-chat meeting times to attend. Course instructors encourage the adolescent to do the homework by mentioning it in the chat sessions, by sending a SMS and by e-mails. During the sessions, course material are introduced by the facilitators and displayed in the chat room using text and images. The user can respond, share experiences, and ask questions

	of peers. Emoticons can be used to express feelings. Users can freely and anonymously consult a 'mood expert' via the e-mail service.
Maya	Via an online gaming system users interact with a narrative hero story in which a young teenager "Maya" models how to deal with negative thoughts, inter-personal problem solving and changes her behaviours. Users make decisions within the game to move the story forward. A scoring system is used to track and provide automated feedback cues about Maya's situation. After completing the game users are provided with personalized post-game feedback that reinforce positive behaviour, presents questions for self-reflection and provides additional psycho-education.
MoodGym	The user creates a login and password. On the website the user is introduced to six distinct characters that form the basis of examples and discussion. Each character has a specific way of dealing with stressful situations, which are explored in the program. Anxiety and depression quizzes are completed at the beginning of each module and at the end of the program. The user's answers to the exercises and quizzes are recorded in a personal workbook, which they can access at any time throughout the program.
Mood Helper	The website includes self-guided online tutorials. The site has four sections "Measure You Mood" (self-assessment); "Facts About Depression (psychoeducation)"; "Journal" (permits the user to record their thoughts and concerns in a private online journal or diary and electively publish entries for viewing by other website users) and "Thought Helper" (the user can type a personal negative or irrational thought into a text search box). The program searches a predefined list of more than 300 common negative thoughts and displays the best matches and a list of several possible realistic counter-thoughts.). The user is prompted to return to the website every few days. Website algorithms process these tracking data to generate personalized feedback regarding the association between mood and pleasant activity level. The website identifies those activities that the user reports occurring infrequently and suggests solutions.
Mood Mechanic Course	Free online program. The user receives weekly phone or email support form a therapist. The course lessons each include (1) reading a lesson on a series of slides; (2) downloading a practice guide that provides skill-building activity ideas; (3) reviewing real-life stories from other people; and (4) contact with the therapist to answer questions and provide support.
OIPE	In this Online Interactive Patient Education program the therapist augments in-person therapy with online materials. The materials lead the user to play pre-recorded scenarios, complete problem-solving activities online, set up homework activities and goals, and review infographics on therapeutic concepts.
Rebound	Within the online platform users complete a card sorting activity to

	identify personal areas of strength. The platform records this information to tailor the content and reinforce users to focus on areas of strength. Within the online social networking space users direct themselves to therapeutic “steps” each requiring approximately 20 minutes to complete. There are 56 steps available. Online peer and clinician moderators are able to recommend steps to youth but ultimately youth are free to explore. Steps are thematic and are not required to be completed in any particular order. Embedded in each step are unique behavioural experiments whereby users employ therapeutic content in their offline world (practice) and then record results online.
SPARX	An interactive fantasy game that utilizes both first person instruction and a three dimensional interactive game in which the user chooses an avatar and undertakes a series of challenges to restore the balance in a fantasy world dominated by GNATs (Gloomy Negative Automatic Thoughts). At the beginning and end of each module, the user interacts in the first person with a “guide,” who puts the game into context, provides education, gauges mood, and sets and monitors real-life challenges, equivalent to homework. If a user does not improve, they are prompted to seek help from their referring clinicians.
Thiswayup	In the online component, the user follows a cartoon-based storyline of teenagers with anxiety or depression solving real life problems. Each lesson takes 40 minutes. After completing the online session, the user is given a handout from the teacher to stimulate discussion and reinforce the information.
